# In Vitro Cytotoxicity and Morphological Assessments of GO-ZnO against the MCF-7 Cells: Determination of Singlet Oxygen by Chemical Trapping

**DOI:** 10.3390/nano8070539

**Published:** 2018-07-18

**Authors:** Fozia Shaheen, Muhammad Hammad Aziz, Mahvish Fatima, Muhammad Ajmal Khan, Faisal Ahmed, Riaz Ahmad, Muhammad Ashfaq Ahmad, Turki S. Alkhuraiji, Muhammad Waseem Akram, Rizwan Raza, Syed Mansoor Ali

**Affiliations:** 1 Department of Physics, Government College (GC) University, Lahore 54000, Pakistan; foziashaheen@gcu.edu.pk; 2 National Synchrotron Radiation Laboratory, University of Science and Technology China (USTC), Hefei 230026, China; 3 Department of Physics, COMSATS Institute of Information and Technology, Lahore 54000, Pakistan; ajmalkhan@ciitlahore.edu.pk (M.A.K.); maahmad@ciitlahore.edu.pk (M.A.A.); rizwanraza@ciitlahore.edu.pk (R.R.); 4 School of Life Sciences, University of Science and Technology China (USTC), Hefei 230027, China; 5 Department of Physics, University of Lahore, 54000 Lahore, Pakistan; mahvish.fatima@phys.uol.edu.pk; 6 CAS Key Laboratory of Magnetic Materials and Devices, Key Laboratory of Magnetic Materials and Application Technology of Zhejiang Province, Ningbo Institute of Materials Technology and Engineering, Chinese Academy of Sciences (CAS), Ningbo 315201, China; 7 Department of Chemical Engineering, COMSATS Institute of Information and Technology, Lahore 54000, Pakistan; faisalahmed@ciitlahore.edu.pk.; 8 The Centre for Advanced Studies in Physics (CASP), Government College (GC) University, Church Road, Lahore 54000, Pakistan; 9 King Abdulaziz City for Science and Technology—KACST, Nuclear Science Research Institute, P.O. BOX 6086, 11442 Riyadh, Saudi Arabia; khuraiji@kacst.edu.sa; 10 Institute of Fundamental and Frontier Science, University of Electronic Science and Technology of China, Chengdu 610054, China; 11 Department of Physics and Astronomy, College of Science, King Saud University, Riyadh 11451, Saudi Arabia; mansoor_phys@yahoo.com

**Keywords:** graphene oxide (GO), cytotoxicity, reactive oxygen species (ROS), cellular morphology

## Abstract

Graphene-based materials have attracted considerable interest owing to their distinctive characteristics, such as their biocompatibility in terms of both their physical and intrinsic chemical properties. The use of nanomaterials with graphene as a biocompatible agent has increased due to an uptick in dedication from biomedical investigators. Here, GO-ZnO was characterized by X-ray diffraction (XRD), field emission scanning electron microscopy (FESEM), ultraviolet-visible (UV-Vis) spectroscopy, energy dispersive X-ray analysis (EDAX), and Raman spectroscopy for structural, morphological, and elemental analysis. The toxic extent of GO-ZnO was noted by a methyl-thiazole-tetrazolium (MTT), while cellular morphology was observed towards the MCF-7 cells using an inverted microscope at magnification 40×. The cytotoxic effect of GO-ZnO investigated the cell viability reduction in a dose-dependent manner, as well as prompted the cell demise/destruction in an apoptotic way. Moreover, statistical analysis was performed on the experimental outcomes, with *p*-values < 0.05 kept as significant to elucidate the results. The generation of reactive oxygen species (ROS) demonstrated the potential applicability of graphene in tumor treatment. These key results attest to the efficacy of GO-ZnO nanocomposites as a substantial candidate for breast malignancy treatment.

## 1. Introduction

In the modern era, nanotechnology and nanomedicine can yield a new, targeted method that promises substantial improvements in cancer treatment [1,2]. In the emerging area of nanomedicine, graphene-based materials (GBMs) are the absolute most investigated nanomaterials [3]. The natural uses of graphene and graphene-related nanomaterials have drawn significant consideration for mainstream researchers in light of their incredible potential for use in bio-imaging [4], antibacterial [5], parasitology [6], biosensing [7], and drug delivery [8]. Graphene nanomaterials have drawn an enormous amount of interest and research enthusiasm due to their physical properties, such as their exceptionally large surface area, good electronic conductivity, and low electrical distortion noise [9,10]. These nanomaterials have now been produced in a wide range of structures with adaptable physical as well as biomedical attributes [11]. Accordingly, in vitro cytotoxicological examinations are required in order to evaluate the systematic assessments of the biocompatibility of graphene-related materials.

Graphene and graphene-based nanocomposites have been shown to induce apoptotic/necrotic prudence caused by reactive oxygen species (ROS) as well as devastation of cell membranes, and may be a biomarker as an anticancer agent [12,13]. Conversely, graphene decreases the mitochondria membrane potential (MMP) and, thus, protracted degrees of intracellular ROS, which actuate the mitochondria subsidiary apoptotic pathway. Graphene thus expands the oxidative stress and metabolic action associated with the revamping functionality inside cells [13,14]. In addition, GO is the vital graphene family member for in vitro cytotoxic study. Adsorption capability of GO can yield dose-dependent cytotoxicity, which may be reduced by incubation with 10% fetal bovine serum (FBS) [13,14]. Horváth assessed the graphene oxide (GO) and reduced graphene oxide (RGO) toxicity in human lung cells and RAW 264.7 phagocytes. They ascertained that cells treated with 0.0125–12.5 μg/cm^2^ of GO or RGO evinced dose-dependent cytotoxicity, which was due to the intracellular ROS generation [15]. Zhang et al. explored folic acid-conjugated NGO (FA-NGO) loaded with two anticancer drugs in targeted delivery, and prominent toxicity to breast cancer cells was exhibited [16].

ZnO is an exceptional helpful semiconductor oxide with a wide direct band gap (3.37 eV) at room temperature. Zinc oxide nanoparticles are always contributing a key part as biocompatible agents and their toxic quality is associated with oxidative stress and cell cycle detainment. Therefore, ZnO is renowned to be a biocompatible material because of the particle suspension in tissue culture media; toxic consequences for living cells have been demonstrated in many studies [17,18]. Interestingly, ZnO nanoparticles likewise prompt cytotoxicity by means of the overproduction of ROS as well as the liberation of Zn^2+^ ions. Therefore, the cytotoxicity could similarly be because of free zinc ions ensuing from the extracellular degradation of ZnO nanoparticles [19]. Conversely, graphene is auxiliary-biocompatible in light of the fact that the living cells may well hold and proliferate on graphene sheets [20]. Though ZnO nanoparticles are usually considered toxic in nature, their lethality can be soothed to make the material biocompatible and nontoxic via appropriate surface variation with graphene sheets [20,21,22].

The potential part of graphene nanocomposites in a couple of applications consists of the assessment of their toxic features in human cellular models [23,24]. Due to their unique properties, various investigations have been performed to evaluate the toxicities of graphene nanoparticles (NPs) on the different cellular framework in in vitro studies and test strategies [23,24,25]. The incidence of breast malignancy has increased continuously in the last decade. GO and ZnO are for the most part used as a component of restorative research, especially in toxicological examinations, and contain a couple of properties that can accept an overarching part in cancerous cell treatment [26,27,28]. However, few data concerning the application of graphene-based nanomaterials to breast malignant cells have been reported, and no study has yet systematically investigated the cytotoxic effects and morphological assessment mechanisms of GO-ZnO on MCF-7 cells. Therefore, in our work, GO-ZnO was tested on an MCF-7 cell line to explore the optimal uptake/absorbance. The present study investigated the cytotoxicity of GO-ZnO by examining cell viability and lactate dehydrogenase (LDH) activity on cancerous breast cells. In addition, nanocomposites depicted morphological variations against MCF-7 cells, which were also a vital impact on the cellular contrivance concerning its therapeutic effects. The possible mechanisms were evaluated by ROS, an apoptotic detection assay, and inverted microscopy. This study provides detailed information about the cytotoxic effects of GO-ZnO on cancerous breast cells and offers a sound basis for the clarification of its toxicity mechanisms. In addition, GO-ZnO were ruminated regarding their competency to generate the singlet oxygen through chemical trapping processes (DPBF; 1,3-diphenylisobenzofuran). Moreover, this study can also be helpful in advanced stage exploration of graphene nanocomposites in nanomedicine.

## 2. Material and Methods

### 2.1. Graphene Oxide (GO)-ZnO Nanocomposites’ Preparation

Hummer’s method was used to prepare the GO through the oxidation of the graphite powder. Ten milliliters of GO and 50 mL of 1 M zinc nitrate Zn(NO_3_)_2_·H_2_O were dispersed in 20 mL of ammonia under vigorous stirring. The mixture was transferred to an autoclave and heated at 120 °C for 10 h. The obtained composite was then centrifuged to separate the nanocomposites from the mixture at 8000 rpm for 5 min, and washed with ethanol several times to purify the nanocomposites and adjust the pH value about 7. Finally, the resulting product was dried overnight in a vacuum oven at 70 °C [29].

### 2.2. Cell Culturing and Labeling (MCF-7, Breast Cancer Cell Line)

Breast carcinoma cells cultured and refined in a T-75 flask (NuncWiesbaden, Wiesbaden, Germany) comprised of a 10 mL solution (Eagle’s Minimum Essential medium EMEM + 10% (*v*/*v*) fetal bovine serum (FBS) + 1% bovine insulin) of a complete growth medium. Moreover, for a proper tie-up, the cells were arranged for incubation for 24 h at 37 °C. Afterwards, MCF-7 cells were sub-refined twice or thrice in seven days and cleaned with 0.25% (*w*/*v*) trypsin to accomplish 76–80% confluence [21,29]. Subsequently, breast cancer cells were gleaned and hatched in 96-well plates with density of 1 × 10^5^ cells/well. Furthermore, GO-ZnO nanocomposites were incubated with varying concentrations (10, 20, 40, 60, 80, and 100 µg/mL) at 37 °C for 24 h with 10% FBS and 5% CO_2_. GO-ZnO doses were set in 96-well plates with rising concentrations (10–100 μg/mL), while the remaining column was used as the control group [30,31]. MCF-7 cells not exposed to GO-ZnO nanocomposites served as the control in each experiment.

### 2.3. In Vitro Cellular Cytotoxicity methyl-thiazole-tetrazolium (MTT) Assay

The cytotoxic assessment of MCF-7 cells was evaluated with MTT (3-(4,5-dimethyl thiazol-2yl)-2,5-diphenyl tetrazolium bromide). The medium from the wells was evacuated after incubation. At that point, MTT (6 mg/mL) was incorporated into each well. The cells were spread into 60 μL of DMSO (dimethylsulfoxide) dissolvable by washing the media again. The specimens’ absorbance spectra were distinguished by taking a wavelength of 595 nm via a microplate reader [32]. The cell viability (% of control) is expressed as the percentage of (OD_test_ − OD_blank_)/(OD_control_ − OD_blank_), where OD_test_ is the optical density of the cells exposed to the GO-ZnO sample, OD_control_ is the optical density of the control sample, and OD_blank_ is the optical density of the wells without MCF-7 cells [33].

### 2.4. Membrane Integrity

Lactate dehydrogenase (LDH) leakage determined the cell membrane integrity of cancerous human breast cells in vitro by using the TOX7 assay kit. Various concentrations (0–100 μg/mL) of GO-ZnO were exposed to the MCF-7 cells for 24 h in a 96-well plate, and a 100 μL mixture of lactate dehydrogenase (LDH) was then added into each well. The microplate reader at a wavelength of 510 nm was used to record the optical density of color generation after 4 h of incubation [34].

### 2.5. Reactive Oxygen Species Fluorescence

Intracellular ROS generation was identified utilizing the non-fluorescent compound CMH2DCFDA (Invitrogen Partnership Carlsbad, Carlsbad, CA, USA). After the compound passes through, the cell membrane experiences demobilization by esterases, which creates the nonfluorescent CMH2DCF, which responds to reactive oxygen species within cells. Subsequently, cells were treated with various concentrations of GO-ZnO at 37 °C for 12 h in humidified air with 5% of CO_2_ [35].

### 2.6. Cell’s Morphological Analysis

For 24 h, breast cancer cells were plated into six-well plates at a density 1 × 10^4^ cells/well and incubated among the GO-ZnO composites of varying concentrations. The morphological assortments were evaluated via inverted phase contrast microscopy to show the distractions instigated by the GO-ZnO nanoparticles in MCF-7 cells as shown in Figure 6.

### 2.7. Cell Mortality Assay

The cell mortality assessment was done using trypan blue assay [36]. Breast cells were plated into 6-well plates at 1 × 10^5^ cells/well and incubated for 24 h. Moreover, diverse concentrations (20, 40, 60, 80, and 100 µg/mL) of GO-ZnO were introduced into the cells in the culture medium. Untreated cultured cells were taken as a control group. The supernatant mixture of detached cells was centrifuged at 1200 rpm/min for 5 min. Afterwards, the residue was then added to a 700 µL trypan blue solution and again dispersed. Cells were counted after 5 min staining by a cytometer. The cell mortality (%) was expressed as percentage of number of the dead cells/number of the total cells.

### 2.8. Apoptosis Detection Assay

Induction of GO-ZnO nanocomposites produced morphological variations in MCF-7 cells when gauged by the double stain process using the PI/AO (propidium iodide/acridine orange) [37]. MCF-7 cells treated with GO-ZnO after 12 and 24 h of incubation. Cells were examined by a fluorescence microscope (Zeiss, Germany). Cells were then centrifuged for 10 min, and the supernatant was discarded. Cells were then colored with 10 μL of a fluorescent dye mixture, and the freshly stained MCF-7 cells were placed on the glass slides. These glass slides were observed and examined under a fluorescence microscope.

### 2.9. Exposure of Singlet Oxygen by Chemical Trapping

To attain the release of singlet oxygen (^1^O_2_) into the solution by the GO-ZnO nanocomposites, 1,3-diphenylisobenzofuran (DPBF) has been done as explained in previous studies [38,39]. An ethanol solution of 3 mL with 50 μM diphenylisobenzofuran (DPBF) and 100 μg/mL of the GO, ZnO NPs, and GO-ZnO or methylene blue (MB) solution was activated in a quartz cuvette in dark. The trials were attained by exposing samples to the sunlight filtered afar a shortpass infrared filter (<550 nm). The solution absorbance was calculated at 410 nm, after 30 s for 3 min with a Nano Drop 2000 (Thermo Fisher Scientific, Waltham, MA, USA). All experiments showed the reduction in absorbance prompted by photobleaching of DPBF. The ^1^O_2_ quantum yield triggered due to nanocomposites (GO, ZnO NPs, and GO-ZnO) into the aqueous solution was measured by taking MB as a standard by the formula below:

(1) $$\Phi_{\Delta }^{b}=\frac{\Phi_{\Delta }^{a}}{I^{a}}I^{b}.$$

The ^1^O_2_ quantum yield of the nanoparticles and MB are demonstrated by $\Phi_{\Delta }^{b}$ and $\Phi_{\Delta }^{a}$, respectively, which was estimated by rose bengal (RB) as a standard (*Φ*_RB_ = 0.75 in H_2_O [39]). ‘*I^b^*’ and ‘*I^a^*’ represent the slope of nanocomposite and MB, respectively, which expresses the time for lessening in DPBF absorption.

### 2.10. Characterization

The morphological variations of GO-ZnO were determined by field-emission scanning electron microscope (FE-SEM, Nova^TM^ NanoSEM 450, Hillsboro, OR, USA), while UV-Visible spectroscopy was achieved by a UV-visible spectrophotometer (UV-2450, Shimadzu, Kyoto Prefecture, Japan). Energy dispersive X-ray analysis (EDAX, Ametek, Inc., Mahwah, NJ, USA) was achieved to evaluate the elemental study as it was combined with SEM. Raman spectra were noted in the range of 400–2000 cm^−1^ via Ramboss Raman spectrometer (Renishaw, Charfield, UK) by argon laser functioning at 514 nm. X-ray diffraction (XRD) analysis of GO-ZnO was attained on a PANalytical X’Pert-PRO (Tokyo, Japan). The average crystallite size was assessed by Scherer’s formula as shown below:

*D_β_* = 0.89*λ*/*β*cos *θ*. (2)

Here, *λ* is the incident wavelength of radiations, and *β* gives the full width at half maximum (FWHM).

### 2.11. Statistical Analysis

The experimental outcomes were assessed as the mean ± standard deviation using Excel software (Microsofts, Henderson, NV, USA) for three independent experiments. Afterwards, the results were calculated via Student’s *t*-test, and a *p*-value less than 0.05 was considered statistically significant. A graph of linear calibration was plotted to check the cell viability of GO-ZnO (concentration: 10–100 µg/mL), representing the linearity [35].

## 3. Results and Discussion

### 3.1. X-ray Diffraction (XRD) Analysis

**Figure 1:**
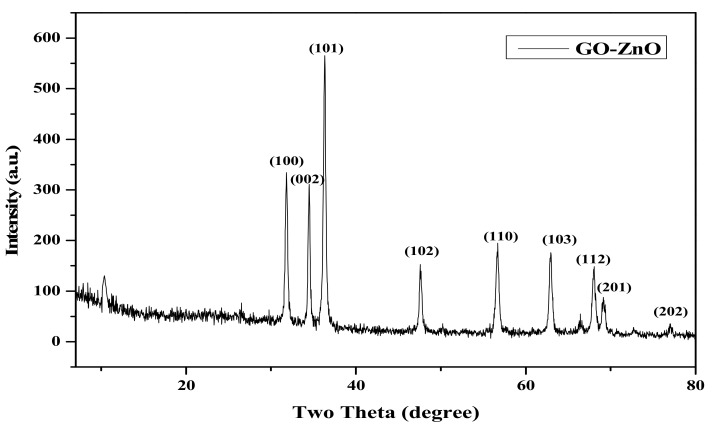
X-ray diffraction (XRD) structure of nanocomposites.

The XRD pattern of GO-ZnO nanocomposite is shown in Figure 1. The peaks of GO-ZnO nanocomposite can be clearly observed at 31.84°, 34.52°, 36.40°, 47.65°, 56.70 °, 62.84°, 67.97°, 69.20°, and 77.00°, which correspond to wurtzite hexagonal structure with the JCPDS No. 36-1451[40]. The crystalline size of ZnO calculated by Scherer formula was 56 nm. Due to the high crystallinity of ZnO, the diffraction of GO in the GO-ZnO nanocomposite is weak and could be seen at 2*θ* = 10.4°, suggesting that the perfect oxidation and the interlayer distance of the graphene sheet is 8.76 Å [40,41].

### 3.2. Scanning Electron Microscopy (SEM) and Energy Dispersive X-ray Analysis (EDAX) of Graphene Oxide (GO)-ZnO

**Figure 2:**
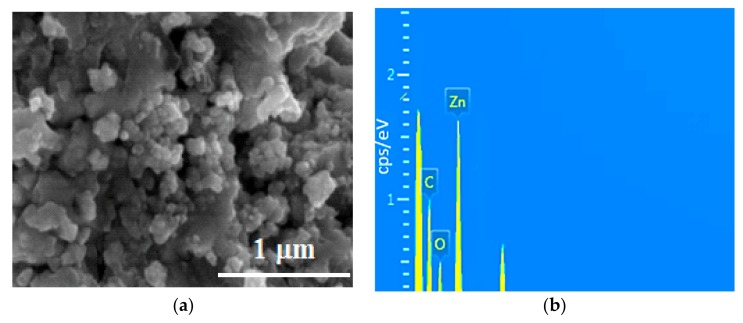
(**a**) scanning electron microscopy (SEM) of graphene oxide (GO)-ZnO nanocomposites; (**b**) energy dispersive X-rays (EDAX) of GO-ZnO.

To study the morphology of the GO-ZnO composite, a FESEM image was taken, and this image is shown in Figure 2a. It can be clearly observed that the nanoparticles of ZnO were dispersed on graphene oxide sheets and that some ZnO nanoparticles were agglomerated. The FESEM image shows that the average nanoparticle size of ZnO was about 62 nm. For the elemental composition of the GO-ZnO composite, the energy dispersive X-ray spectroscopy (EDAX) used and spectrum is given in Figure 2b. The characteristic peaks C, O, and Zn were observed in the EDAX spectrum, and the atomic and weight ratios are 25.71, 23.64, and 50.65 (wt %), respectively [40,41,42,43].

### 3.3. Raman Spectroscopy and Ultraviolet-Visible (UV-Vis) Analysis

**Figure 3:**
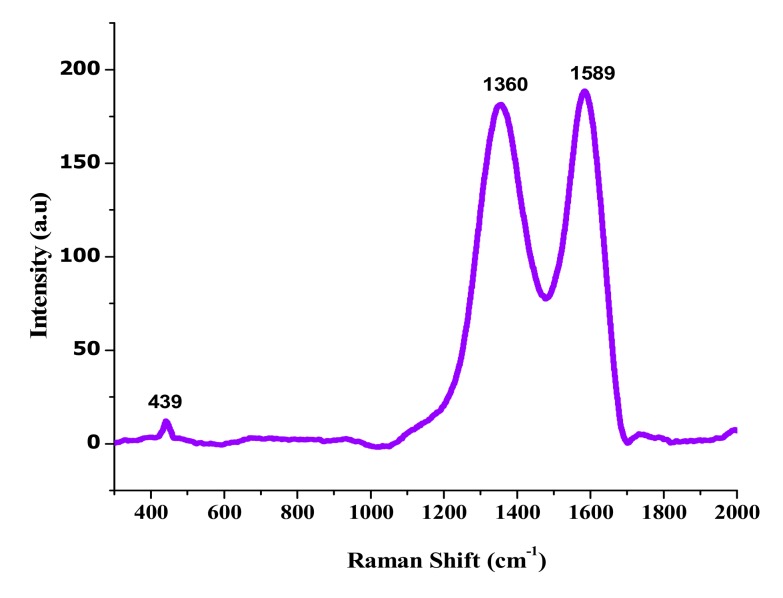
Raman spectra of GO-ZnO nanocomposites.

**Figure 4:**
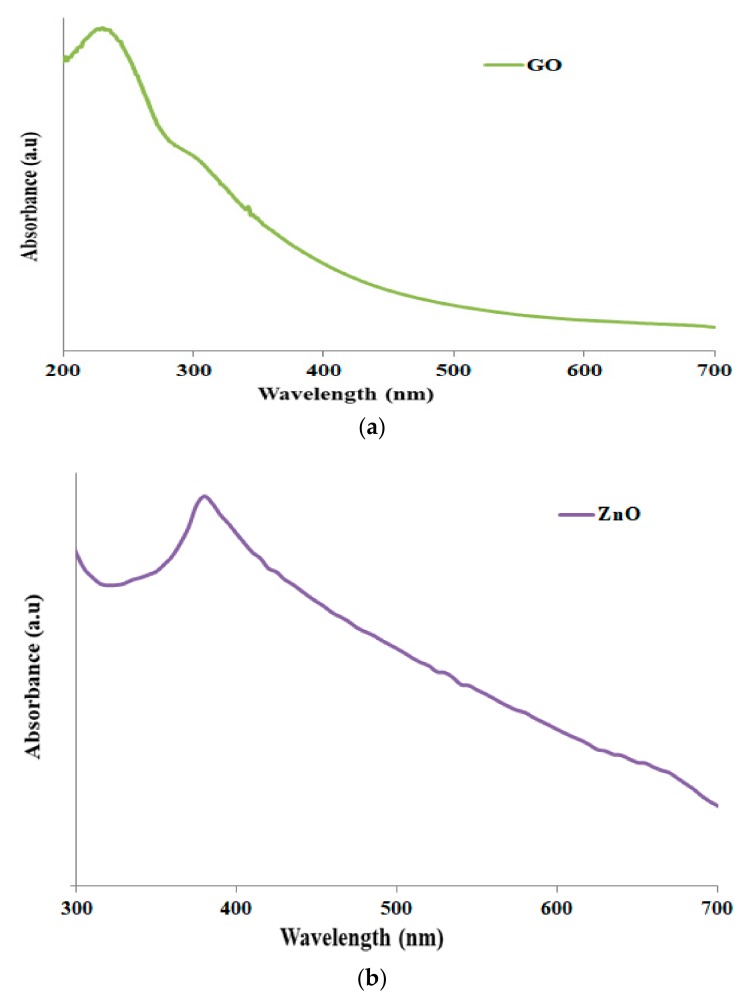

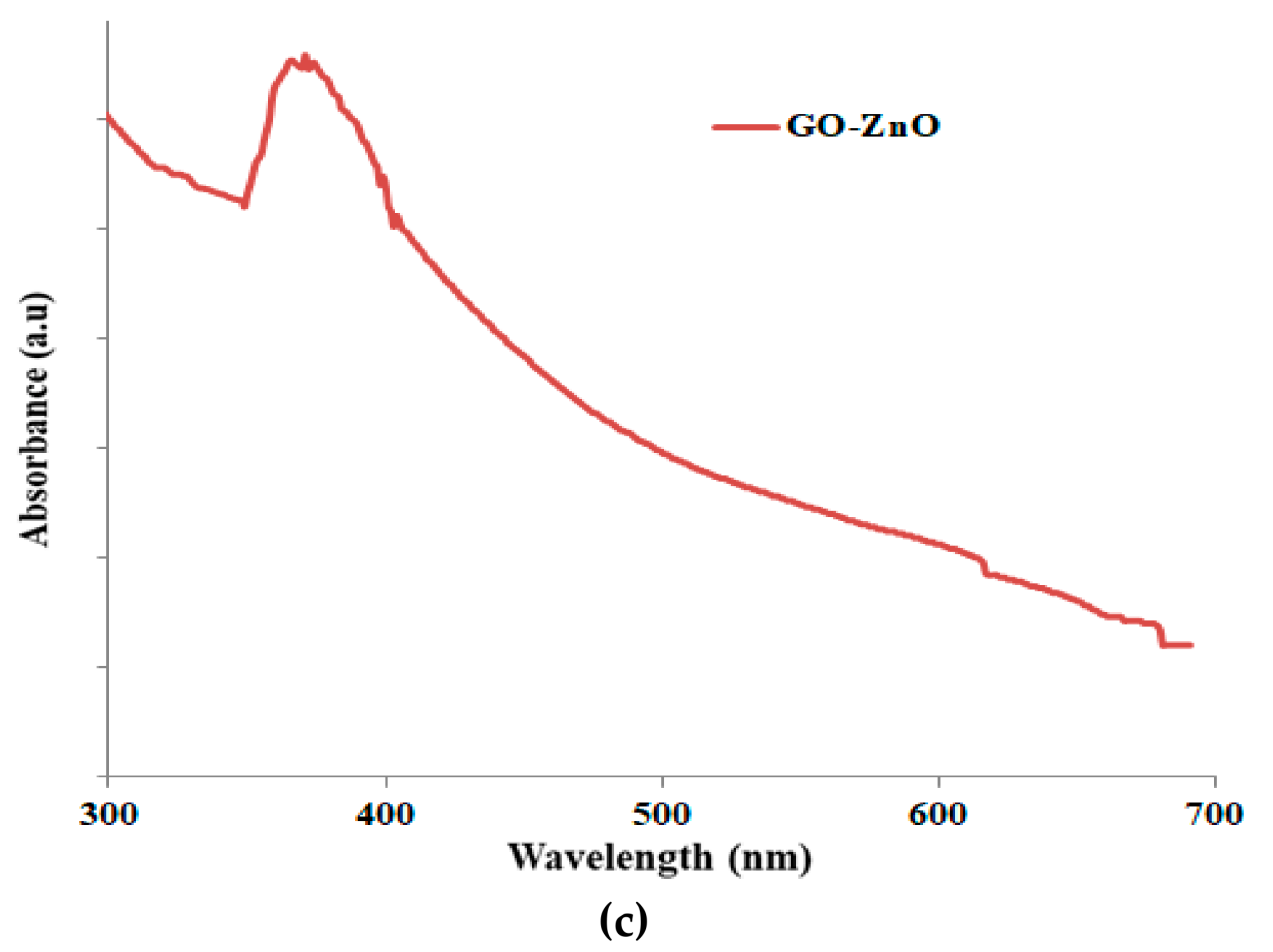
Ultraviolet visible absorption spectra of (**a**) GO; (**b**) ZnO; (**c**) GO-ZnO nanocomposites.

Raman spectra of GO-ZnO mixtures are shown in Figure 3. The peak at 439 cm^−1^ corresponds to the E2 (high) vibration mode of ZnO. The peak at 1350 cm^−1^ is the D band from the vibration of defect states in graphene sheets, and the peak in the vicinity of 1589 cm^−1^ is assigned to the G band vibration of carbon materials. The G peak position of the original GO is located at 1597 cm^−1^. After reduction, the G peak shows a measurable red shift to 1589 cm^−1^, which is also an indicator of GO reduction [44]. As described in Figure 4, ZnO nanoparticles ensured that the absorption peak was at a wavelength of 380 nm in Figure 4b, while the GO shows the obtained peak at 220 nm in Figure 4a. However, the attained GO-ZnO in Figure 4c demonstrates an extreme absorption peak at 351 nm after internalization of ZnO NPs with GO, which resulted in rapid electron transfer and increased transition energy [43,44,45].

### 3.4. Cellular Uptake and Cytotoxicity of GOZnO towards MCF-7 Cells

**Figure 5:**
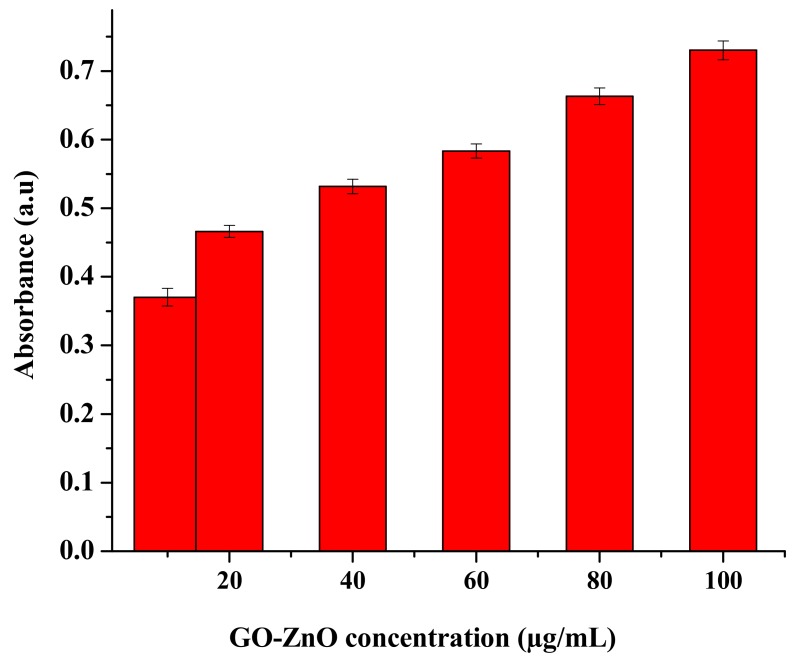
Absorbance versus concentration of GO-ZnO nanocomposites.

Graphene composites have particular physicochemical effects and are practical for a few prospects. Their biological properties in organisms will eventually determine their purpose. The most appropriate biomedical employments of graphene-ZnO nanocomposites have been exalted to various applications such as antibacterial properties and nanocarriers for controlled stacking on medicating conveyance transport as an anticancer operator [46]. For cytotoxicity evaluation, a systematic study was accomplished to analyze the deadliness of GO-ZnO toward MCF-7 cells, as well as to decide the probability of cell destruction. In this study, we attempted to take the absorbance using GO-ZnO after 24 h, as clarified in Figure 4. The recent study revealed the optimal density/absorbance of the utility of GO-ZnO in a breast cancer cell line. Furthermore, it was exposed by expanding the concentration of GO-ZnO, the mean absorbance of the said nanocomposites prolonged to 0.6 a.u., as shown in Figure 5. These consequences demonstrated the dependence of the noteworthy loss of cell viability and reactive oxygen species (ROS), which portrayed the prominent malignant cell/tissue damage via cell necrosis/apoptosis alone. These effects confirmed that GO-ZnO had extraordinary biocompatibility with MCF-7 cells.

**Figure 6:**
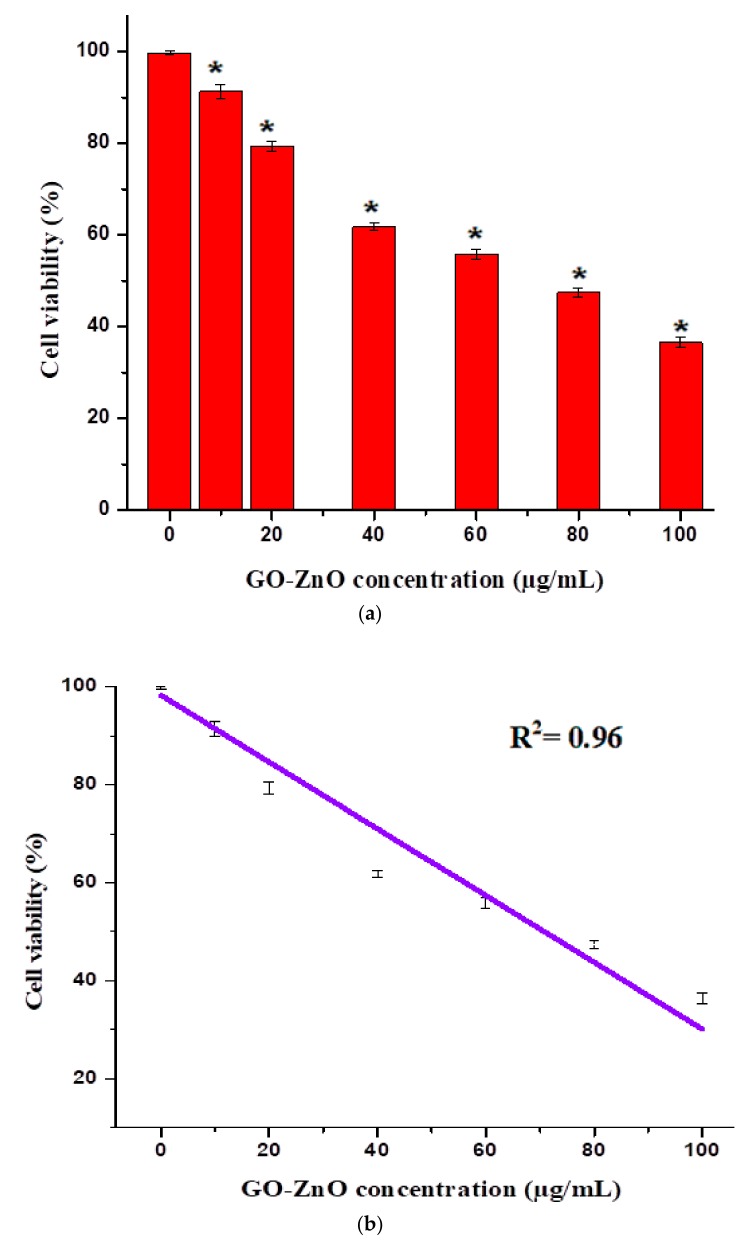
(**a**) cellular viability of GO-ZnO treated MCF-7 cells, *t*-test (* *p* < 0.05); (**b**) linear calibration plot of GO-ZnO vs. cell viability.

The cytotoxicity impacts of GO-ZnO nanocomposites were assessed by employing the MTT assay. GO showed nearly 13% cell viability in breast cancer cells in the dose-dependent approach [47,48]. In contrast, the fluorinated graphene oxide (FGO) at the concentration of 576 μg/mL revealed no cytotoxicity to breast carcinoma [49]. Graphene oxide cytotoxicity has moreover been clarified in the HBI.F3 human neuronic cells and BEAS-2B human lung cells, displaying the decrease in cell viability at a dose of 10–100 μg/mL. In addition, both early and late apoptosis of cells were enhanced when matched to the control [50]. The cytotoxicity of erythrocytes and skin fibroblast according to Liao et al. [51] enhanced with the concentration of GO. In other study aspects, it was exhibited by increasing the concentration of GO [52], the cytotoxic effect on HepG2 cells amplified. It is observed that the synergetic effects between ZnO nanorods and reduced graphene oxide (RGO) sheets enhanced the antioxidant properties towards the human embryonic kidney cells (HEK293), which in turn produced the excellent cytotoxicity effects. The reason is that zinc ions dispersed on the RGO sheets enabled intimate contact with cancerous cells and trapped cells to their demise [53]. In another study, GO-FA-ZnO with increasing doses from 0 to 100 μg/mL decreased cell viability to 45%, 31%, 25%, and 19% as compared to the control [54]. The impact of graphene oxide on the viability using the MTT assay was also investigated by de Marzi et al. with the same cell line [55]. GO was used for two different chip sizes (1.32 μm and 130 nm) and a range of concentrations (10, 50 and100 μg/mL). After 24 h of incubation with both types of GO, the results showed insignificant reduction in the viability of the A549 cells [56]. In comparison, the toxicity of GO-Ag to breast cancer cells may also be synergistic, which maximized the interaction among the cells. It is seen that breast cancer cells were treated via diverse concentrations (10–100 µg/mL) of GO-Ag for 24 h, which, in turn, indicated the decrease in cell viability in a dose-dependent manner [57]. Therefore, in comparison to earlier studies, the said nano-structural material was used to determine the possibility of cell death (due to chemical reaction or mechanical stress/trauma). Cancerous breast cells had diverse concentrations ranges from 10 to 100 µg/mL in GO-ZnO for 24 h, as found in Figure 6, demonstrating the noteworthy reduction in cell viability in a dose-subjected way. Cell viability was decreased to 90%, 79%, 62%, 55%, 47%, and 37% for MCF-7 cells at the concentrations of 10, 20, 40, 60, 80, and 100 µg/mL, respectively. Figure 6a shows a decrease in cell viability near 37% at 100 µg/mL, which is significant (* *p* < 0.05, *t*-test). It is also clearly synchronized with cellular uptake as long as absorbance increases (as shown in Figure 5). Cell viability loss also increases gradually, which is the uniqueness of the current conducted experiment. Nevertheless, cytotoxicity results of GO-ZnO nanocomposites carried out two simple principles for a definite anticancer agent, i.e., tumor particularity and minimal toxicity towards normal cells. Furthermore, explained data clarified that the cellular viability loss was in a dose-dependent manner [51,52]. Therefore, the toxicity of GO-ZnO to cancerous breast cells may also be synergistic, which exploited the interaction between cells. The interactive toxicity importance of GO-ZnO towards MCF-7 cells can contain distractions of cell membrane and cause oxidative stress. Additionally, the linearity signifies the importance of the regression equation analysis (*Y* = 94.54 − 0.6131X) from the data achieved (*n* = 7) by the GO-ZnO cell viability vs. concentration relationship in Figure 6b. The intercept had a value of 94.54 with a negative slope of 0.6131. The value for the *R*^2^ = 0.96. Therefore, statistical outcomes supported the precision of the experimental evidence.

### 3.5. GO-ZnO Effect on Membrane Integrity

**Figure 7:**
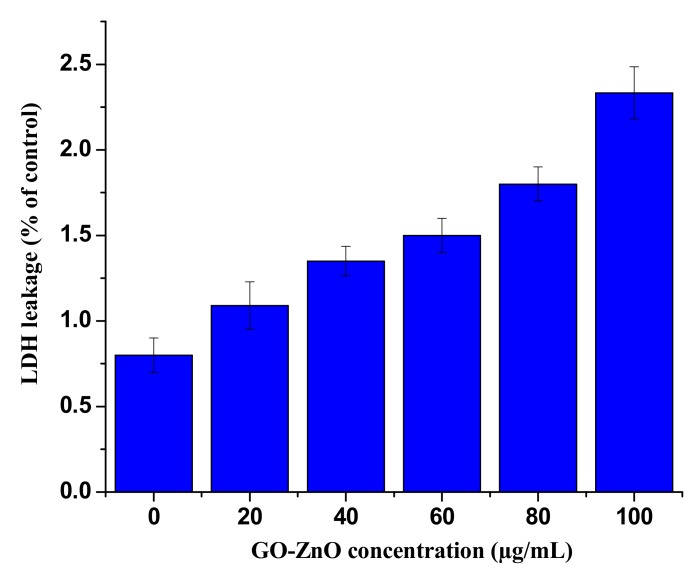
Lactate Dehydrogenase (LDH) release (%) in MCF-7 cells following incubation with GO-ZnO nanocomposites after 24 h. Treated groups exhibited the statistically significant differences from the control group, as measured by the Student’s *t*-test (*p* < 0.05).

Apoptosis occurred as a result of cell membrane damage, and the root cause of these phenomena is the release of LDH, a soluble cytosolic enzyme, into the extracellular medium. It is generally known as an indicator of lytic cell death. In our study, LDH activity was measured to observe the effect of GO-ZnO on membrane integrity by treating cancerous breast cells for 24 h. The results proved that the influence of GO-ZnO on the cell membrane integrity of MCF-7 cells responds in a dose-dependent manner and increases as concentration increases, as shown in Figure 7. This was observed in slightly amplified LDH activity at doses of 40–80 µg/mL, as compared to the control cells. In another study, it is determined that graphene nanoribbons MCF-7 cells showed the extreme LDH release at higher concentration as compared to the positive control after 24 h, which is almost the same after 72 h [58,59].

### 3.6. Morphological Variations and Cell Mortality on MCF-7 Cancerous Cells by GO-ZnO

**Figure 8:**
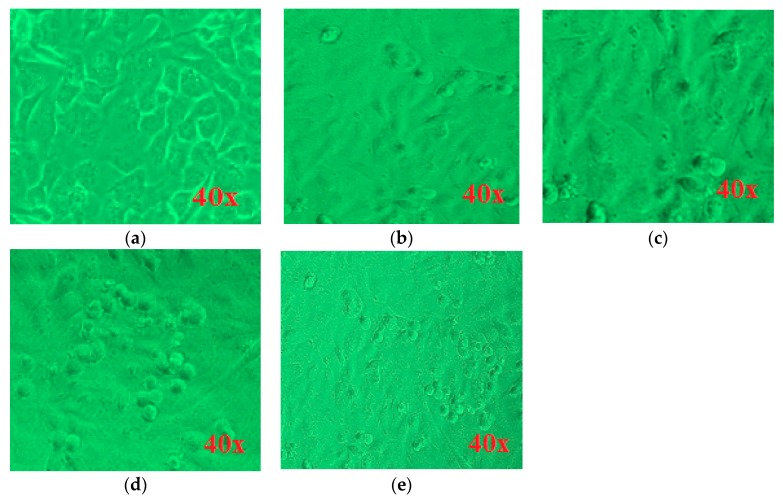
GO-ZnO induced morphological variations in MCF-7 cells at magnification 40× (**a**) control; (**b**) 20 µg/mL; (**c**) 40 µg/mL; (**d**) 80 µg/mL; (**e**) 100 µg/mL.

**Figure 9:**
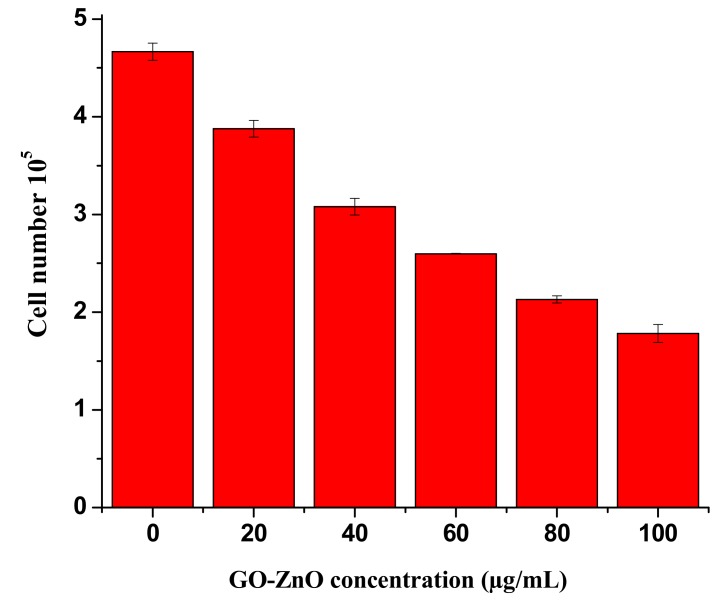
The outcomes of GO-ZnO represent the cell mortality of MCF-7 Cells. Treated groups displayed the statistically significant differences from the control group, as measured by the Student’s *t*-test (*p* < 0.05).

Cellular viability is verified by evaluating the effect of GO-ZnO nanocomposites on the cellular morphology of MCF-7 cells. Previous studies have expressed the utilization of graphene-based derivatives prompted apoptosis in cancerous cells. Hence, we verified that the accumulation of the GO-ZnO nanocomposite to MCF-7 cells created the definite prominent effect on the cellular morphology [47]. The cell viability results of products were also confirmed by photomicrographs of the cancerous breast cells incubated in the existence of GO-ZnO nanocomposites for 24 h, as illustrated in Figure 8. The largest number of viable cells was exhibited in the control group. The control MCF-7 cells turn out as large adherence cells with an epithelium with long arms and inconclusive cell boundaries. MCF-7 cells when investigated with GO-ZnO appeared to be different from those of the control shown in Figure 8a. At various concentrations of GO-ZnO, such as 20, 40, 80, and 100 μg/mL, as found in Figure 8b–e individually in MCF-7 cells, a decreased amount of cells and a great impact on cellular morphology were perceived. Considerably at higher level concentrations, treated MCF-7 cells looked like fewer covenants with contracted arms. In the same way, Jaworski et al. [60] indicated the obvious cytotoxicity of GO and RGO in the glioma cells. Similarly, a reduced quantity of cells and a significant effect on the cellular morphology were observed in A2780 cells when labeled with GO. The GO-treated cancerous breast cells seemed marginally separate from those of the control group [60]. Although viability assays displayed cell mitochondria activity, mortality analysis specified cell death. In our study, trypan blue assay was used to monitor cell mortality. Cell mortality is expressed by the ratio of dead cells to total cells. A noteworthy cell death could be observed upon treatment with GO-ZnO, as matched with untreated cells in Figure 9. Cheng et al. showed that, even with a biopolymer at a high concentration of 100 µg/mL, functionalized RGO exhibits an ultralow hemolysis ratio and good compatibility in human endothelial cells [61].

### 3.7. Reactive Oxygen Species (ROS) Generation

**Figure 10:**
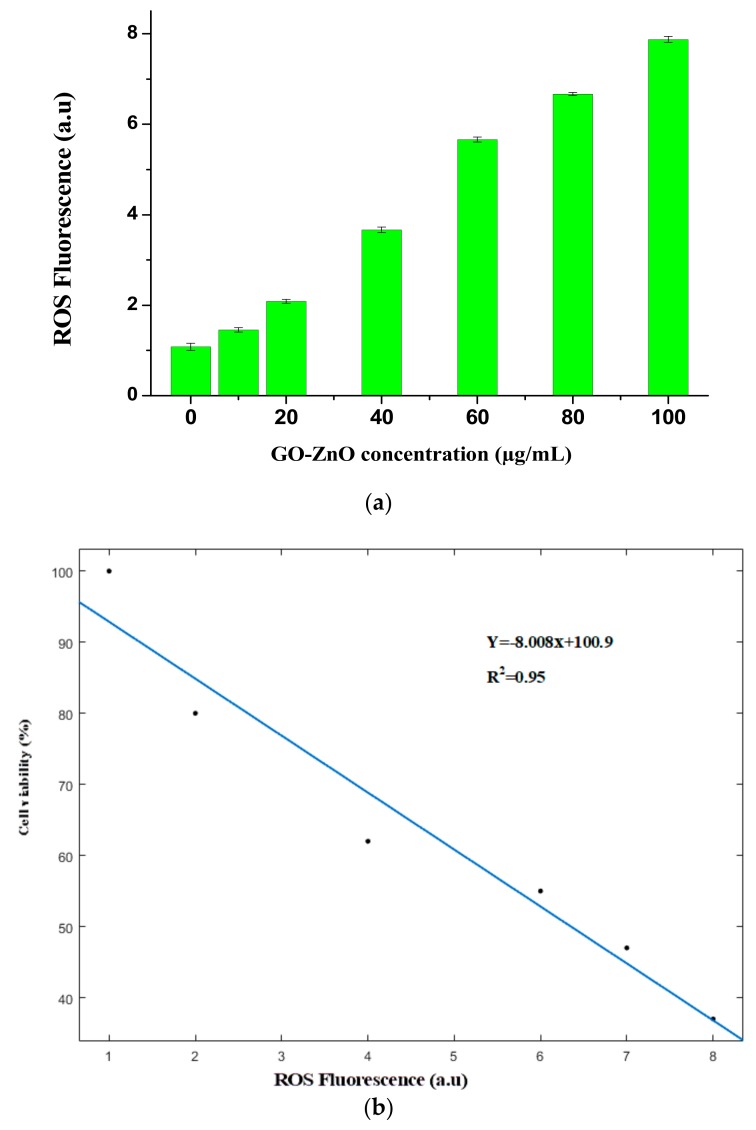
(**a**) GO-ZnO liberated Reactive Oxygen Species (ROS) Fluorescence in breast cancerous cells; (**b**) a significant negative correlation between ROS and Cell viabiity.

Numerous investigations have described the prominence of ROS in cytotoxicity. ROS is one of the suggested toxicological mechanisms of different nanomaterials comprising graphene ROS expansion is the process for cell-belting down influence, such as cell apoptosis or cell necrosis [62]. Moreover, ROS concentrates on mitochondria, which prompts cellular apoptosis by means of vascular blockade [63]. To explore the outcomes of the GO-ZnO nanocomposite on ROS generation via diverse doses (10–100 µg/mL), the results obviously showed that GO-ZnO strongly affects ROS production when matched to the control. As is evidently found in Figure 10a, a consistent escalation in the fluorescence of ROS outcome was noticed. Furthermore, we also perceived an inverse linear relationship between the ROS and cell viability as seen in Figure 10b. These consequences revealed that GO-ZnO liberated ROS and detained the cell sequence by discretion of the MCF-7 cell growth through promoted oxidative stress, which could be valuable for the development of effective graphene-associated derivatives, particularly for use in biomedicine [63,64].

### 3.8. Apoptotic Detection Assay

**Figure 11:**
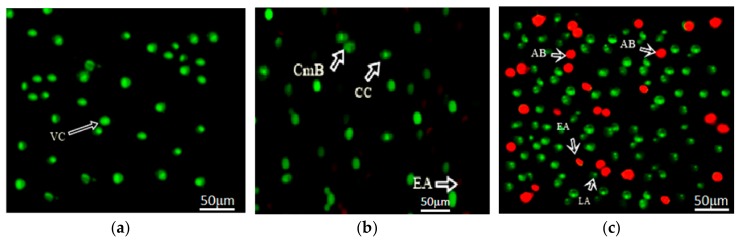
Fluorescent micrographs of (acridine orange/propidium iodide) AO/PI double stained MCF-7 cells that were treated with GO-ZnO nanocomposites (400× magnification). (**a**) untreated cells presenting normal cell structures; (**b**) treated MCF-7 cells after 12 h incubation; (**c**) MCF-7 cells after 24 h incubation. **CmB**: Membrane blabbing; CC: Chromatin condensation; VC: Viable cells, EA: Early apoptotic cells, LA: Late apoptotic cells, and AB: Apoptotic body.

In addition, the analysis in Figure 11 showed that apoptosis was induced by GO-ZnO against MCF-7 cells after 24 h of incubation while Figure 11a represented the fluorescent micrographs of untreated MCF-7 cells giving normal cell structures. Afterwards, morphology of cells was studied for untreated and treated cells. It was observed that the membrane blabbing appeared with the chromatin condensation after 12 h, as confirmed in Figure 11b. Furthermore, early apoptosis with nuclear margination after 24 h were occurred within the cells. Cell death ensued and apoptotic cells appeared, and late apoptotic response was studied in some cells even after 24 h, as observed in Figure 11c.

### 3.9. Liberation of Singlet Oxygen (^1^O_2_)

**Figure 12:**
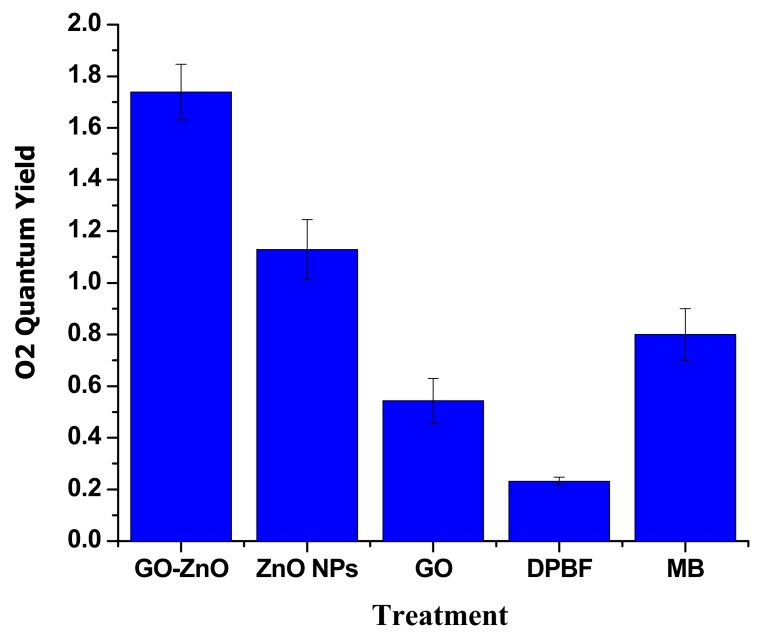
Quantum yield of singlet oxygen of GO-ZnO, ZnO and GO in contrast to MB.

The liberation of ^1^O_2_ into aqueous solution was assessed ultimately via the DPBF assay. DPBF responds irretrievably with ^1^O_2_, instigating a decline in its absorption intensity at 410 nm. The diverse nanoparticles (100 µg/mL) were assorted in the DPBF solution and, upon illumination, absorption was calculated over a period of time [38,39]. As revealed in Figure 12, the quantum yield of DPBF saw a slight increase in ^1^O_2_ production, but MB created a noteworthy growth in the ^1^O_2_ phases. GO also contributed with a momentous surge in singlet oxygen levels, but its ^1^O_2_ level was slightly smaller than MB. ZnO created the comparable instigation of singlet oxygen that was considerably greater than the GO alone. A substantially more significant origination in ^1^O_2_ generation was observed for GO-ZnO.

## 4. Conclusions

In this experimental investigation, GO-ZnO was synthesized by the hydrothermal process and characterized using multiple techniques, such as XRD, FESEM, EDAX, and UV-Vis spectroscopy. Cellular uptake, cytotoxic assessments, and morphological analysis at numerous concentrations showed that GO-ZnO created a significant toxic impact on MCF-7 cells. In addition, GO-ZnO was found to have a cell-killing mechanism at higher doses (60–100 µg/mL). The optimal time of incubation was set to 24 h when GO-ZnO was able to localize in the cell organism, and loss in cell viability was recorded in the MCF-7 cellular model in a dose-dependent manner. GO-ZnO nanocomposites were also found to induce cytotoxicity and oxidative stress in MCF-7 cells, as made evident by ROS production and LDH assay. Furthermore, a significant reduction in cell viability (up to 63%) showed the dependency of ROS liberation and noticeable cancerous cell destruction by cell necrosis/apoptosis (via prompting oxidative stress). As a result, apoptosis response with nuclear margination were ensued inside the MCF-7 cells after 24 h. Hence, it was determined that GO-ZnO nanocomposites are encouraging for biomedical application. Moreover, this study provokes advanced imminent experimental research for the development of graphene-based functional materials for biological applications.
